# Stellate Cell Activation and Imbalanced Expression of TGF-*β*1/TGF-*β*3 in Acute Autoimmune Liver Lesions Induced by ConA in Mice

**DOI:** 10.1155/2017/2540540

**Published:** 2017-01-29

**Authors:** Liyun Wang, Lei Tu, Jinping Zhang, Keshu Xu, Wei Qian

**Affiliations:** ^1^Division of Gastroenterology, Shandong Provincial Qianfoshan Hospital, Jinan, Shandong, China; ^2^Division of Gastroenterology, Union Hospital, Tongji Medical College, Huazhong University of Science and Technology, Wuhan, Hubei, China; ^3^Division of Neurology, Shandong Provincial Qianfoshan Hospital, Jinan, Shandong, China

## Abstract

*Objective*. To study the pathogenic feature of liver injury, activation of hepatic stellate cells, and dynamic expression of TGF-*β*1/TGF-*β*3 to reveal their role in liver injury induced by ConA.* Methods*. Mice were randomly divided into control group and ConA treatment group. ConA (20 mg/kg) was injected through vena caudalis in ConA treatment group; the controls received the same volume of saline injection. After injection for 2 h, 8 h, 24 h, and 48 h, animals were terminated. Blood, liver, and spleen were harvested. Liver function and histopathology were studied. *α*-SMA, vimentin, TGF-*β*1, and TGF-*β*3 were detected.* Results*. After ConA injection, liver damage started to increase. Expression of *α*-SMA, vimentin, TGF-*β*1, and TGF-*β*3 was significantly enhanced; all above indicators reached peak at 8 h; but from 24 h after ConA injection, TGF-*β*3 expression began to decline, while the TGF-*β*1/TGF-*β*3 ratio at 48 h was significantly lower than control.* Conclusion*. (1) Autoimmune liver injury induced by ConA showed time-based features, in which the most serious liver lesions happened at 8 h after ConA injection. (2) Early activation of HSC and imbalance expression of TGF-*β*1 and TGF-*β*3 existed in ConA-induced acute autoimmune liver injury, which may be associated with liver dysfunction and the mechanisms of progression to fibrosis.

## 1. Introduction

Autoimmune hepatitis (AIH) is caused by the disorder of self-immune system, which takes the liver cells as their target cells and leads to damage of mainly parenchymal cells in the liver. Although the pathogenic initiating events are unknown, AIH is considered to be a CD4^+^T cell- mediated disease [1]. If left untreated, the disease may rapidly progress to liver fibrosis, and quite often cirrhosis has occurred in patients with AIH at the first visit to the hospital [2]. Thereby we speculate that there may be some certain factors linking immune responses to liver fibrosis, which prompts the early and rapid origination of cirrhosis caused by immune response in AIH.

ConA is a polyclonal mitogen that can induce specific acute liver damage by activating T lymphocyte. Many lineages of mice are susceptible to ConA to produce hepatitis. ConA-induced hepatitis is T cell-dependent. Single injection of ConA can lead to infiltration of mainly CD4^+^ lymphocytes, macrophages, and other inflammatory cells in the liver parenchyma, causing release of a variety of cytokines and inflammatory mediators induced by specific liver injury [3, 4]. ConA-induced acute liver injury animal model may most closely resemble human autoimmune hepatitis due to the involvement of T cell immunity [5, 6] and therefore is thought to be a good model for human autoimmune hepatitis, providing a useful tool for studying the development and treatment of autoimmune hepatitis.

It is well documented that HSC activation is the key pathological process in the initiation of liver fibrosis. In the process of liver injury, after activation and proliferation, HSC stimulated by the lesion can show fibroblasts and/or smooth muscle cell characteristics, so they are also known as myofibroblast-like cells (MFBLC) [7]. In addition, accumulating evidences suggest the close relationship between HSC activation and immune response; it was identified to be professional liver-resident antigen-presenting cells (APC) [8, 9], whereas more previous studies also showed that activated HSCs could significantly inhibit T cells responses and induce apoptosis of T cells [10–12]. And resent research demonstrated that hepatic stellate cells could increase the immunosuppressive function of natural Foxp3+ regulatory T cells [13]. HSC also induces Treg production in a variety of ways, which showed its function of immune regulation [14, 15]; additional studies also showed that HSC surface molecules B7-H1 expression increased, which induced alleviating of autoimmune liver damage [16]. However, it is not clear whether HSC activation participates in the mechanism of AIH and the possible influencing factors of that.

Transforming growth factor-*β* family is a multifunctional growth factor superfamily involved in immune response, regulation of cell proliferation, differentiation, and extracellular matrix formation. The TGF-*β* family is composed of three known members (TGF-*β*1, TGF-*β*2, and TGF-*β*3) in mammalian species [17], of which TGF-*β*2 has extremely small content in the human body. A variety of blood cells such as T lymphocytes, B lymphocytes, monocytes, osteoblasts, and platelets have the capacity to secrete the nonactivated TGF-*β* [18]. Studies have found that TGF-*β*1 and TGF-*β*2 may promote the formation of collagen, yet wound-healing experiments revealed that TGF-*β*1 and TGF-*β*2 cause fibrotic scarring responses, while TGF-*β*3 induces a scar-free response [19]. In addition, our previous studies also have shown that TGF-*β*3 presents antagonistic role of TGF-*β*1 and TGF-*β*2; at the cellular level and experimental disease models, TGF-*β*3 could inhibit the gene expression of TGF-*β*1 and showed significant inhibition of hepatic fibrosis [20].

TGF-*β* is also a major kind of immune regulatory factors, the most studied TGF-*β*1 mainly as a class of immunosuppressive factor has been confirmed to participate in regulating the occurrence of a variety of inflammations [21]. Activation of TGF-*β*1 not only can enhance and regulate the function of monocytes but also can prevent proliferation and activation of T cells and B cells, additionally inhibiting macrophage maturation and activity; it also showed inhibition of NK cells and lymphokine-activated killer cells (LAK cells) and the further production of proinflammatory cytokines, while TGF-*β*1 also plays an important role in the inflammatory process [22]. In addition, the role of TGF-*β*1 in the pathogenesis of acute AIH has also been confirmed; the mice's lack of TGF-*β*1 gene on T cells exhibited more susceptibility to experimental autoimmune liver disease. There was also a strong expression of TGF-*β*1 in the serum and liver tissues of patients with AIH, which may be related to disease activity [17]. In humans, TGF-*β*1 is the most abundant circulating isoform in plasma. Recently, more studies focused on the role of TGF-*β*3 in immunity; several recent lines of evidence suggest a role for TGF-*β*3 in the pathogenesis of autoimmune diseases [23], which most importantly include the producing of Th17 [24], while indirect evidence suggested that TGF-*β*3 plays an important role in immune regulation. In experimental autoimmune encephalomyelitis (EAE) model of mouse, the induction of EAE was associated with high expression of TGF-*β*1 mRNA and low expression of TGF-*β*3 mRNA [25]. Resent study also reported that CD4+CD25LAG3+ Tregs (LAG3+ Treg) regulate humoral immunity and lupus disease in MRL-Faslpr/lpr mice via TGF-*β*3 production [26]. These above results suggest that TGF-*β*s have bifunctional roles in the immune system, whereby they regulate both proinflammatory and anti-inflammatory activities.

The aim of this work was to investigate the role of HSC activation and the level of TGF-*β*s in acute autoimmune liver lesions induced by ConA in mice and to reveal the possible mechanism and association of inflammation and liver fibrosis in AIH model.

## 2. Materials and Methods

### 2.1. Animals

Female Kunming mice, aged from 7 to 9 weeks and weighed between 28 and 32 g, were originally obtained from the animal center of Tongji Medical College, Huazhong University of Science and Technology. They were housed in cages in a quiet, warm environment away from strong light. The mice colonies were screened and determined to be pathogen-free.

### 2.2. Experiment Design

The mice were randomly assigned to the control group and experimental group; to induce autoimmune liver injury, mice were subjected to intravenous injections of ConA (20 mg/kg weight, Sigma, USA) or saline as the control. Mice from each group were sacrificed 2 h, 8 h, 24 h, and 48 h after injection, respectively; at each time point, 8 mice were anesthetized in the ConA group, while 4 mice in the control group were anesthetized in each parallel point. Cardiac blood was collected, and liver samples were harvested. Whole blood was centrifuged (3000 r/min, 10 min); the supernatant is serum. Serum and liver tissue were stored in −70°C for subsequent assay.

### 2.3. Liver Function Test

Serum samples were sent to Union Hospital, Huazhong University of Science and Technology Laboratory, for detection of indicators of ALT, AST, albumin (*A*), and globulin (*G*).

### 2.4. The Liver Index and Spleen Index

The liver and spleen samples were immediately weighed after being separated. Spleen index = spleen weight (g)/body weight × 1000; liver index = liver weight (g)/body weight × 1000.

### 2.5. HE Staining of Liver and Histology

The tissues were paraffin-embedded using routine protocols and stained with hematoxylin and eosin. Sections were assessed blindly.

### 2.6. Immunohistochemical Staining

Paraffin-embedded, formalin-fixed liver tissue (4 *μ*m) was incubated with anti-*α*-SMA antibody (1 : 200, Boster, China) and anti-vimentin antibody (1 : 200, Boster, China) overnight at 4°C after blocking endogenous peroxidase activity with 0.3% H_2_O_2_, followed by incubation with secondary antibody for 30 mins after washing off free first antibodies with PBS; then autoradiography was taken with DAB (3,3′-diaminobenzidine) reagent followed by counterstaining with hematoxylin. Negative blank controls were set during the staining, in which the first antibody was omitted but replaced by PBS. Scores were made according to the level and range of color: no positive color as was considered as 0 points; range of positive staining less than 26% was considered as 1 point; 26%–50% was considered as 2 points; 51%–75% was considered as 3 points; and more than 75% was recorded as 4 points.

### 2.7. Enzyme-Linked Immunosorbent Assay (ELISA)

Cardiac blood was collected and placed in tubes free of pyrogen and endotoxin. After centrifugation with speed of 1000 ×g for 15 minutes, serum samples were harvested and stored at −70°C for use. TGF-*β*1 and TGF-*β*3 were determined in the serums using commercially available enzyme-linked immunosorbent assays (ELISAs; R&D Systems, Minneapolis, MN, USA), according to the manufacturer's instructions. The sensitivity of the assays was <15 pg/mL.

### 2.8. Western Blot

Protein was extracted and separated by SDS-polyacrylamide gel electrophoresis and was then transferred to polyvinylidene ethylene membranes, blocked from the nonspecific antibody binding sites with non-fat milk powder (concentration of 0.5%) at room temperature for 2 h, and then polyclonal antibodies against *α*-SMA (1 : 200, Boster, China), vimentin (1 : 200, Boster, China), TGF-*β*1 (1 : 200, BioVision, USA), TGF-*β*3 (1 : 100, Abcam, USA), and GAPDH (1 : 5000, Boster, China) were added. This was followed by incubation overnight at 4°C, followed by incubation with a secondary antibody (1 : 5000) for 2 h, and then autoradiograms were taken with ECL reagent in the chamber. Finally, imaging analysis was carried out by an automated electrophoresis gel imaging system.

### 2.9. Data Presentation and Statistical Analysis

We used SPSS 19.0 statistical software for analysis, and the data were displayed as mean ± SME. After variance and homogeneity analysis, one-way ANOVA was taken to test the difference among groups. Independent-samples *t*-tests were taken to compare the mean between two groups. *P* < 0.05 was considered to be significant.

## 3. Results

### 3.1. Liver Function of Mice at Each Time Point after Intravenous Injection of ConA (Table 1)

Our study showed that, after ConA injection, serum ALT and AST of mice increased and reached peak at 8 hours after drug administration, which were significantly higher than the controls (*P* < 0.01). 8 hours later after ConA administration, ALT and AST levels gradually fell, yet at 24 h after ConA injection they still remained higher than the control group (both *P* < 0.05), and at 48 h after ConA administration there were no significant differences with the normal control group. Within 48 hours after injection of ConA, serum albumin did not change significantly compared with the control group, yet immunoglobulin of mice 8 hours after injection tended to increase compared with the normal group but did not reach statistical significance.

### 3.2. Changes of Liver Index and Spleen Index at Different Time Points after Intravenous Injection of ConA (Figure 1)

In our study, liver index of control mice was 4.46 ± 0.32; after intravenous injection of ConA, the liver index tended to increase and peaked at 8 h (6.15 ± 1.53). At 24 h and 48 h after ConA injection, the liver index was 5.78 ± 1.49 and 5.24 ± 1.4, respectively, and at the above three time points, liver index was all significantly higher than in the control group (*P* all < 0.01). Liver index at 2 h after ConA injection was 4.97 ± 0.34, with no significant difference compared with the control group (*P* = 0.36). Spleen index of control mice was 1.33 ± 0.18; similar to the liver index, it tended to increase after ConA injection and also reached the peak at 8 h, and, at 2 h, 8 h, 24 h, and 48 h time points after ConA injection, spleen index was 1.59 ± 0.10, 1.93 ± 0.14, 1.73 ± 0.10, and 1.74 ± 0.14, respectively, which was significantly higher than the control group (*P* all < 0.05).

### 3.3. Major Organ Morphology and Histopathology of the Mice of Different Group (Figure 2)

In the normal control mice, hepatic lobular structures were normal, hepatic cord was arranged neatly, and there was no congestion of the central venous; at 2 h after ConA treatment, the liver showed mild congestion and mild infiltration with lymphocyte in portal area and the hepatic cord appeared to be in slightly disordered arrangement, but the hepatic cells' morphology was still normal; 8 h after ConA injection, the liver revealed significant liver congestion, disappearance of the liver cord and sinus, steatosis of the hepatic cells, and infiltration of numerous inflammatory cells within the portal area; 24 h after ConA injection, liver congestion, degeneration of the liver cells, and moderate infiltration of inflammatory cells remained; after 48 h, there was mild degeneration of liver cells and mild infiltration of inflammatory cells in portal area.

### 3.4. Protein Expression of HSC Activation Markers in Liver after Injection of ConA (Figures 3–6)

**Figure 3:**
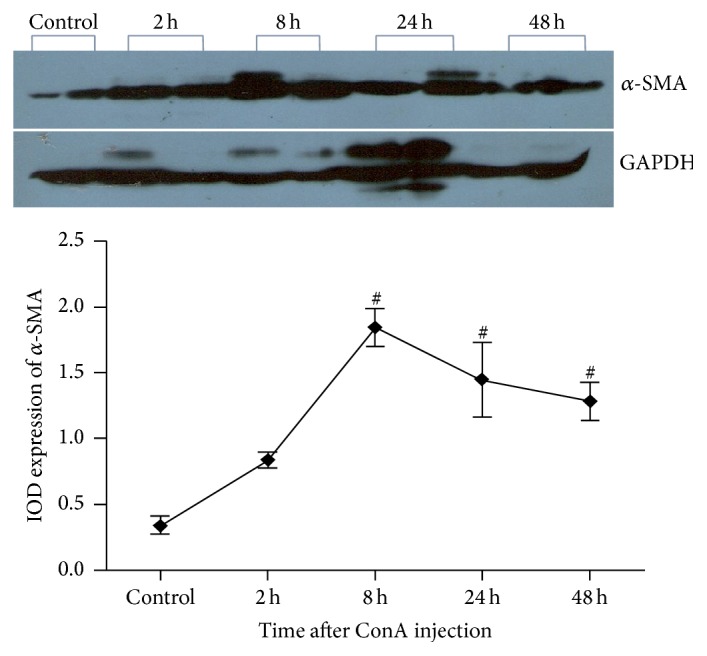
Protein expression of *α*-SMA in liver after injection of ConA. ^#^*α*-SMA protein expression is significantly enhanced compared to the control group at 8 h, 24 h, and 48 h after ConA injection (*P* all <0.01).

In this study, we selected *α*-SMA and vimentin as the markers of HSC activation, both of which are expressed mainly in the cytoplasm of activated HSC. Western blot analysis showed the relative liver expressions of *α*-SMA protein in control mice were 0.34 ± 0.12; 2 h after injection of ConA, relative expressions of *α*-SMA protein were 0.84 ± 0.10, which showed no significant statistical difference compared with the control group (*P* = 0.184). After 8 h, *α*-SMA protein expression rose to 1.84 ± 0.33, which was significantly higher than the controls (*P* = 0.001); it also showed significant increasing trend compared to 2 h time points (*P* = 0.007); relative expressions of *α*-SMA protein at 24 h and 48 h after ConA injection were 1.45 ± 0.36 and 1.28 ± 0.38, respectively, and both were significantly higher than the normal control group (*P* = 0.004 and 0.009); there was no significant difference in *α*-SMA protein expression among 8 h group and 24 h and 48 h group (pairwise comparison, *P* > 0.05) (Figure 3).

**Figure 4:**
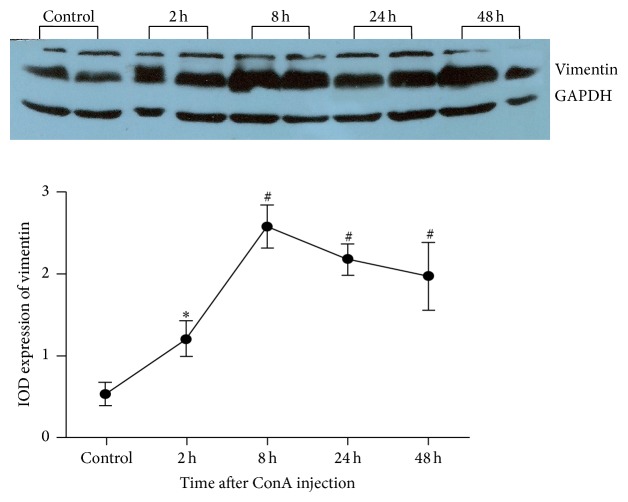
Protein expression of vimentin in liver after injection of ConA. ^*∗*^Vimentin expression was significantly increased compared to the control group at 2 h after ConA injection (*P* = 0.012). ^#^Vimentin expression was significantly enhanced compared to the control group at 8 h, 24 h, and 48 h after ConA injection (*P* all <0.01).

Relative expression of vimentin protein in liver of normal control group was 0.60 ± 0.09, after injection of ConA at 2 h relative expression of vimentin protein increased to 1.3 ± 0.26 and was significantly higher compared with the control group (*P* = 0.012); protein expressions of vimentin at 8 h, 24 h, and 48 h after ConA injection were 2.43 ± 0.52, 2.12 ± 0.48, and 1.97 ± 0.72, respectively, in which there were significant differences compared with the normal control group (*P* all < 0.01) (Figure 4). These results suggest that, after ConA injection, *α*-SMA and vimentin protein expressions were significantly increased in the liver of mice, in which enhancement of vimentin expression appeared earlier than *α*-SMA (Figure 4).

**Figure 5:**
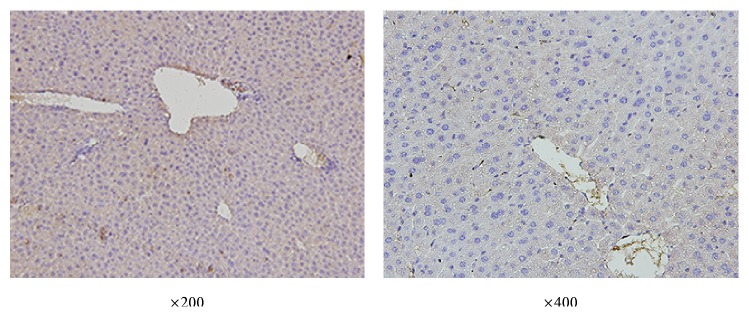
Immunohistochemistry staining of vimentin in liver of control mice.

**Figure 6:**
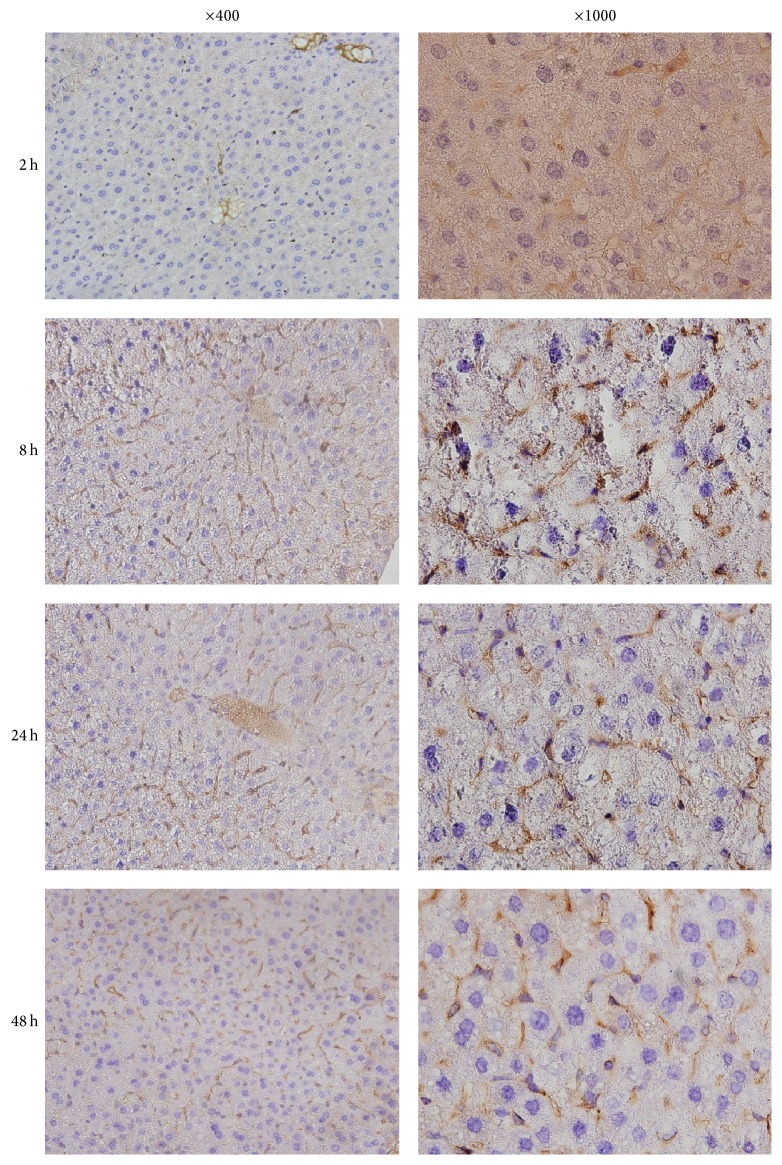
Immunohistochemistry staining of vimentin in liver of mice after ConA injection.

Similar to Western blot results, immunohistochemistry showed that control mice had no significant vimentin positive cells in liver; at 2 h after ConA injection, the mice showed a slight stain in liver, and at 8 h, 24 h, and 48 h after ConA injection, significantly increased positive staining was observed in the liver, among which the strongest stain appeared at 8 h after ConA injection, shown in Figures 5 and 6.

### 3.5. Levels of Serum TGF-*β*1 and TGF-*β*3 in Mice after ConA Injection (Table 2)

As the two main TGF-*β* isoforms, TGF-*β*1 and TGF-*β*3, are important immune regulatory factors, they have the opposite effect in promoting liver fibrosis. Serum TGF-*β* was secreted by a variety of blood cells such as T lymphocytes, B lymphocytes, monocytes, osteoblasts, and platelets, while in inflammatory state, these cells increase secretion of TGF-*β* to regulate the inflammatory responses, so serum level of TGF-*β* may reflect the immune regulatory capacity of the body. ELISA results showed (Table 2) that, after ConA injection, serum concentrations of TGF-*β*1 enhanced since 2 h and reached the peak at 8 h, which was significantly higher than the control group (*P* = 0.031). After 8 h concentrations of TGF-*β*1 decreased gradually to 28.77 ± 8.44 at 24 h, yet they were still significantly higher than the normal control group (*P* = 0.046); 48 h after ConA injection, serum concentrations of TGF-*β*1 reduced to the level of no significant differences with the normal control group. Besides, after ConA injection, alteration of serum TGF-*β*3 concentration showed similar trends to those of TGF-*β*1 before 8 h after ConA injection; there was a slight upward trend before 8 h, at which the peak concentration arrived and was significantly higher than the control group (*P* = 0.025). Since 8 h to 48 h after ConA injection, serum TGF-*β*3 concentration decreased rapidly, but it was still higher than the normal mice at 24 h after ConA injection (*P* = 0.043), yet serum concentrations of TGF-*β*3 reduced to the level of no significant difference with the control group at 48 hours after ConA injection. After ConA injection, the ratio of serum TGF-*β*3/TGF-*β*1 showed similar trend to the changes of TGF-*β*1 and TGF-*β*3, which showed no significant difference compared with the control group from 2 h to 24 h after ConA injection, but the ratio of TGF-*β*3/TGF-*β*1 decreased significantly at 48 h compared with control group (*P* = 0.046).

### 3.6. Protein Expressions of TGF-*β*1 and TGF-*β*3 in Liver of Mice after ConA Injection (Figure 7)

**Figure 7:**
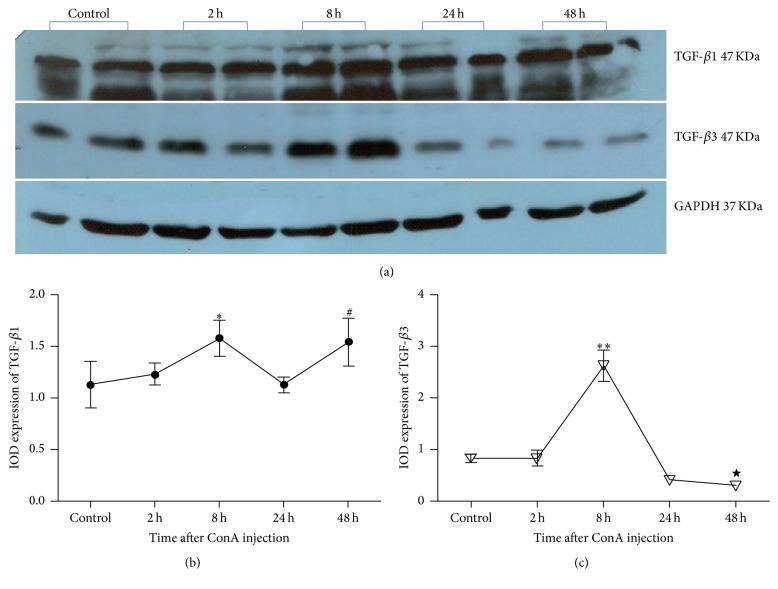
Protein expressions of TGF-*β*1 and TGF-*β*3 in liver of mice after ConA injection. ^*∗*^At 8 h after ConA injection, TGF-*β*1 protein expression was significantly higher than that of the control group (*P* = 0.037). ^#^At 48 h after ConA injection, expression of TGF-*β*1 was significantly higher than that of the control group (*P* = 0.0234). ^*∗∗*^At 8 h after ConA injection, the relative expression of TGF-*β*3 was significantly higher compared with the control group (*P* = 0.000). ^★^At 48 h, TGF-*β*3 protein expression decreased to the level that was significantly lower than the control group (*P* = 0.033).

A large part of expression of TGF-*β*1 and TGF-*β*3 in the liver comes from the secretion of HSC; as both TGF-*β*1 and TGF-*β*3 play an important role in the regulation of HSC activation and liver fibrosis, their expressions perhaps reflect a side of the activation of HSC; moreover, change or imbalance of the ratio of TGF-*β*1/TGF-*β*3 may be involved in the immune response or progression of liver fibrosis. Western blot analysis showed that relative expression of liver TGF-*β*1 protein in the control group was 1.08 ± 0.07; 2 h after ConA injection, relative expression of liver TGF-*β*1 was 1.21 ± 0.22, with no significant difference compared with that of the control group (*P* = 0.597). Yet at 8 h after ConA injection, relative expression of liver TGF-*β*1 protein raised to 1.65 ± 0.20, which was significantly higher than that of the control group (*P* = 0.037). After that, at 24 h and 48 h after ConA injection, relative expressions of liver TGF-*β*1 were 1.41 ± 0.17 and 1.72 ± 0.34, respectively, and the latter was significantly higher than that of the control group (*P* = 0.0234) (Figures 7(a) and 7(b)).

On the other hand, relative expression of liver TGF-*β*3 protein in the control group was 0.83 ± 0.08; 2 h after ConA injection, TGF-*β*3 expression was 0.83 ± 0.15, with no significant difference with the control group (*P* = 0.991). 8 h after ConA injection, the relative expression of TGF-*β*3 protein rose to 2.62 ± 0.30, which was significantly higher compared with the control group (*P* = 0.000). Since 24 h after ConA injection, relative protein expression of TGF-*β*3 showed a downward trend, in which TGF-*β*3 protein expression was 0.41 ± 0.053 at 24 h, and showed a decrease tendency, but there was no significant difference compared with control group (*P* = 0.192); at 48 h, relative TGF-*β*3 protein expression in liver decreased to 0.30 ± 0.05, which was significantly lower compared with the control group (*P* = 0.033) (Figures 7(a) and 7(c)).

### 3.7. Correlation Analysis between Each Two of Serum TGF-*β*1, TGF-*β*3, and TGF-*β*3/TGF-*β*1 and Intrahepatic Expression of *α*-SMA and Liver Transaminase Levels (Table 3)

Highly positive correlations existed between TGF-*β*1 expression and ALT, AST, and *α*-SMA levels (*r* = 0.967, 0.966, and 0.912; *P* = 0.07, 0.007, and 0.018, resp.). And positive correlations were also found between the TGF-*β*3 expression and ALT and AST (*r* = 0.907 and 0.918, resp.; *P* = 0.033 and 0.028, resp.). Yet no significant correlations were shown between TGF-*β*3/TGF-*β*1 ratio and transaminases or *α*-SMA (*P* all > 0.05). Expression of *α*-SMA showed similar trend to that of transaminases, but there was no significant correlation between *α*-SMA and the level of ALT or AST (*P* value was 0.056 and 0.090, resp.).

## 4. Discussion

Autoimmune hepatitis (AIH) is a T cell mediated disease. Little is known about the factors influencing susceptibility to AIH. Compared to other kinds of hepatitis, cirrhosis in AIH occurs earlier and progresses more rapidly. If left untreated, the disease may rapidly lead to liver fibrosis, and cirrhosis quite often is the first presentation of a patient with AIH. ConA is a polyclonal mitogen that can stimulate specific T cell activation and lead to the occurrence of acute liver injury. ConA-induced liver damage is mediated by T cells [4, 27]. As it involves T cell immunity, animal model of ConA-induced acute liver injury has the most similar characters to human autoimmune hepatitis [5, 6]. ConA-induced liver damage is characterized by elevated serum aminotransferase levels and rapid secretion of large amounts of inflammatory cytokines, inflammatory leukocyte infiltration in the liver, necrosis, and apoptosis of the liver cells.

Our study showed the certain time limitation of liver injury and pathological changes during the process of modeling, indicating that the single dose-ConA-induced acute liver injury mediated by excessive immune reactivation may be self-recovered in a short time. No significantly elevated transaminases were observed 2 h after 20 mg/g ConA via tail vein injection, yet 8 h after ConA injection transaminases were found to enhance significantly, which subsequently showed downward trend on 24 h and 48 h after ConA injection, indicating that ConA had a time-dependent effect on liver injury. At the same time, histopathological examination also showed that 8 h is during the most severe period of acute liver injury caused by ConA, when transaminases levels, liver, and spleen indexed also reached the peak. These results were consistent with previous researchers' conclusions. Our results also showed that the spleen index of mice was significantly increased at 2 h after modeling, but there was no increase in the liver index at that time. The results showed that in the liver injury model induced by ConA, spleen pathological changes may precede the occurrence of liver injury, which may also be related to pathological mechanism reported in the previous study [5, 28], which found that the activated T lymphocytes of the spleen would flow to the liver with blood, inducing further activation of macrophages and cytokines and causing liver damage. In this experiment, the level of serum albumin in the model group was not significantly different from those in the normal control group at all time points, suggesting that the time effect of ConA-induced acute liver injury in mice was of short term and the liver reserve function still existed. In the present study, we found that although there was no significant difference compared with the normal control group, the levels of serum globulin in mice showed increasing trend correlated with modeling, which may be related to overactivation of immune response in liver injury.

Most studies suggest that HSC activation is the key pathological process to start hepatic fibrosis; *α*-SMA and vimentin are the marker proteins of HSC activation [29–31]. HSC activation is also related to the chronic inflammation caused by various diseases. However, the role of HSC activation has not been reported in early inflammation stages of AIH, when fibrosis is not yet obviously formed.

TGF-*β* family is a group of cytokines that exhibit a variety of effects of biological functions such as embryogenesis, carcinogenesis, immune response, regulation of cell proliferation and differentiation, and extracellular matrix formation. From the insight of liver fibrosis, previous studies have shown full understanding about the profibrotic TGF-*β*1 and antifibrotic TGF-*β*3. While the other aspect in immune response, TGF-*β*1, is most studied and identified as immune-regulating factor, the role of TGF-*β*3 in immune response gradually attracted interest in recent years, which was initially observed in the pathogenesis of autoimmune diseases, indicating its proinflammatory role [23, 24]. However, recent studies also showed the anti-inflammatory functions of TGF-*β*3. As TGF-*β*1, TGF-*β*3, and HSC all have been shown to participate in both fibrosis and immune response in previous studies, a large part of the secretion of these two TGF-*β* subtypes in the liver is derived from HSC, causing counter-reaction in the HSC and forming an autocrine regulatory network. Furthermore, significantly higher expression of TGF-*β*1 has been suggested by studies in clinical testing of serum and liver tissue in active AIH patients [17], suggesting the role of TGF-*β*1 in early lesions of AIH, while clinical study also found that *α*-SMA expression was significantly increased in the liver of patients with AIH compared with normal control, suggesting the presence of abnormal activation of HSC in liver of patients with AIH [32]. Thus, we speculate that the abnormal activation of HSC may exist in early stage of AIH, which interacts with the unbalanced expression of TGF-*β*1 and TGF-*β*3 and promotes further development of fibrosis.

In this study, acute AIH animal models were established through ConA intravenous injection; at the early time points after ConA injection, protein expressions of HSC activation markers, TGF-*β*1 and TGF-*β*3, were detected. Evaluating the excessive activation of HSC, as well as the imbalanced expression of TGF-*β*1 and TGF-*β*3 in the early stage of the model, could provide a theoretical basis for the mechanism involved in the fibrosis progression in AIH patients.

In the present study, *α*-SMA and vimentin were selected as HSC activation markers, in which the former was considered as the marker of smooth muscle cell, while the latter was considered as fibroblast marker. Our results showed that, at 2 h after ConA injection, no significant difference of *α*-SMA protein expression was found compared with the normal control group, yet the protein expression of vimentin significantly increased compared with the control group at the same time point. The expression of both *α*-SMA and vimentin reached the peak at 8 h after ConA injection, and 24 h and 48 h after injection, their expressions remained at a significantly higher level than the control group. These results suggest that abnormal activation of HSC appears at the early stages after the injection of ConA. The liver function and pathological results showed that the most serious liver injury occurred at the 8-hour time point, combined with the peak expression of HSC activation markers; thus we concluded the relation between HSC activation and the liver injury. Studies have shown that antigen-stimulated T cells and their secreted factors after activation would be strong stimuli for HSC activation [11]; thus we believe that ConA injection-induced T cell activation may be an important factor for the early HSC activation, which also causes liver damage, so it was reasonable of the peak time consistency between liver injury and HSC activation. The results also showed that the higher expression of vimentin appeared earlier than that of *α*-SMA, because vimentin could be expressed in myofibroblasts and fibroblasts after HSC activation, while *α*-SMA was only expressed in myofibroblasts, so it is possible that expression of vimentin may be more likely to increase from a quantitative point of view; also it may possibly be due to the complexity of cellular components in the liver, which may include a certain number of fibroblasts, leading to early increased expression of vimentin. The results also showed that the relative expression of HSC activation marker protein was kept at higher level in the 24 h and 48 h groups compared to control group. Therefore, we believe that early HSC activation of AIH is may be one of the reasons for the rapid progress of AIH cirrhosis.

Our study also showed that the levels of TGF-*β*1 and TGF-*β*3 in serum and liver increased significantly after 8 h, reaching a peak at 8 h and decreasing after 8 h. Yet the expression of TGF-*β*1 protein in the liver at 24 h and 48 h was still higher than that in the control group, while TGF-*β*3 had decreased to the level of control group at 24 h and further decreased to a level lower than control group at 48 h. The results of the first part showed the similar changes in serum aminotransferase levels of mice in the model groups, which indicated that the liver could perhaps achieve self-repair, in which the main mediator may be regulatory T cells (Treg) [11]. Previous studies have reported that B lymphocytes of resting state could be successfully transformed into regulatory T cells (Treg) induced via TGF-*β*3 in vitro [33], while another experiment observed induced CD4^+^CD25^+^ Treg in 2,3,7,8-tetrachlorodibenzo-p-dioxin treated mice, with simultaneous upregulation of the endogenous TGF-*β*3 gene [34]. These results suggest that TGF-*β*3 may play an important role in the regulation of Treg production and maintenance of its activity, which lead to further speculation of the role of TGF-*β*3 in immune regulation. Thus, it is speculated that the early peak expression of TGF-*β*3 may play an important role in the generation and maintenance of Treg in animal models, while Treg would secrete generous TGF-*β* (mainly TGF-*β*1) to achieve the regulation of liver immune disorders and thus to curb excessive activation of the immune response in time. The production of TGF-*β* in AIH may also be an adaptive process by which the body suppresses autoreactive T cells, preventing the exacerbation of AIH and promoting spontaneous remission of the organism. Therefore, the peak expression of TGF-*β*1 at 8 h time point may be the product of immunoregulation of Treg, and the sustained high expression of TGF-*β*1 after 8 h may be the result of its autocrine feedback regulation. The reverse separated expression of TGF-*β*1 and TGF-*β*3 in the late stage of the model may be due to different secretory cells such as Kupffer cells, hepatic sinusoidal endothelial cells, and hepatocytes [35].

TGF-*β*1 and TGF-*β*3 may be partly secreted by HSC, so the peak expression of TGF-*β*1 and TGF-*β*3 may partly result from the peak activation of HSC. On the other hand, activated HSC may be partially responsible for the imbalance of TGF-*β*1 and TGF-*β*3; also the former may react on HSC, forming an interaction network other than HSC. These changes confirm our hypothesis that imbalance of TGF-*β*1 and TGF-*β*3 expression has existed in the early stages of AIH. TGF-*β*3 is confirmed to be antifibrosis factor, while TGF-*β*1 is recognized as promoting factor of liver fibrosis; due to their opposite roles in the formation of fibrosis, the early expression imbalance of TGF-*β*1 and TGF-*β*3 is consequently believed to lead to early and rapid fibrosis formation in the disease.

In conclusion, our study suggested that abnormal activation of HSC was accompanied by imbalance of TGF-*β*1 and TGF-*β*3 expression in the early stage of acute autoimmune liver damage induced by injecting ConA in mice, which can link immune and hepatic fibrosis processes, leading to further switching and progressing of liver fibrosis, which may be one of the mechanisms involved in the easy progressing of liver cirrhosis in patients with autoimmune hepatitis.

## Figures and Tables

**Figure 1:**
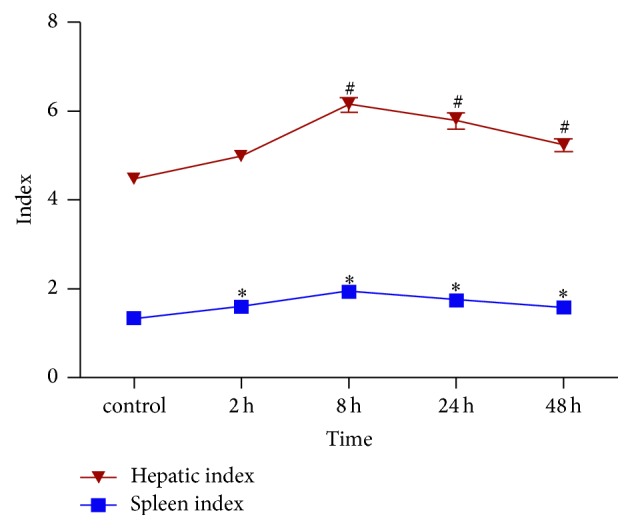
Changes of liver index and spleen index at different time points after intravenous injection of ConA. ^#^The liver index at 8 h, 24 h, and 48 h after ConA injection was significantly higher than in the control group (*P* all <0.01); ^*∗*^spleen index at 2 h, 8 h, 24 h, and 48 h after ConA injection was significantly higher than in the control group (*P* all <0.05).

**Figure 2:**
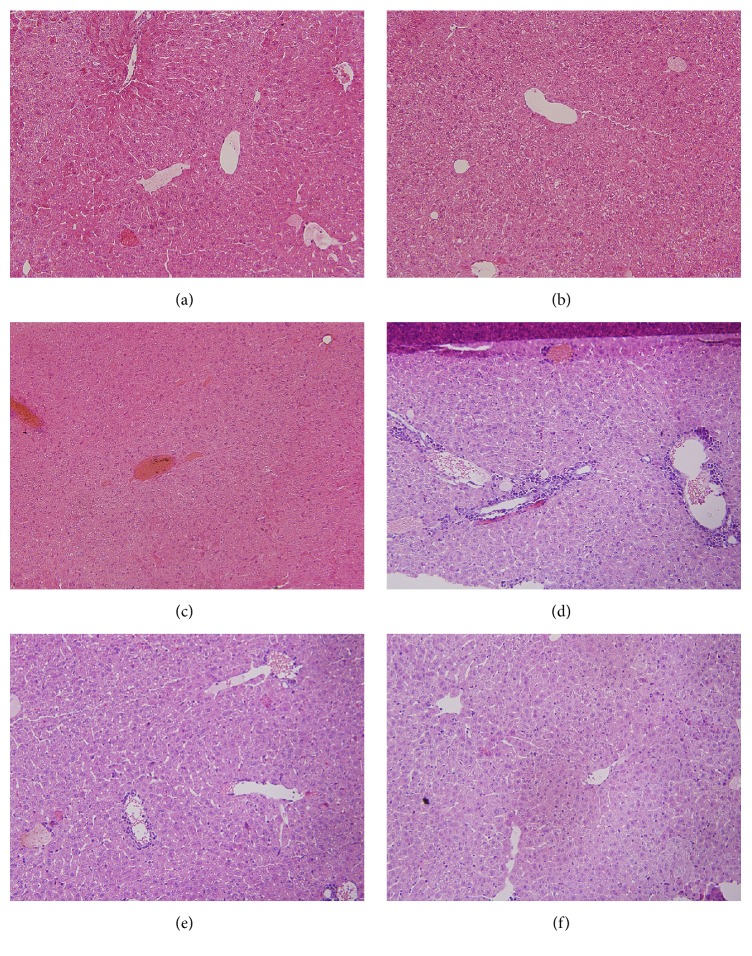
Major organ morphology and histopathology. In the control group, the hepatic lobules of the mice in the control group were normal and clear, the hepatic cord was well arranged, and the central veins were not congested (a). Mild hyperemia occurred in the 2 h group, mild lymphocyte infiltration occurred in the portal area, and mild steatosis occurred in the hepatocytes 2 h after ConA injection (b). 8 h after ConA injection, liver congestion and disappearance of hepatic sinusoid and hepatic cords (c). Cell steatosis and a large number of inflammatory cells' infiltration in the portal area (d) were observed. Liver congestion, hepatocellular degeneration, infiltration of inflammatory cell in the portal area, and hepatic parenchyma (e). Liver damage showed recovering trend 48 h after ConA injection, with no significant inflammatory infiltration in the portal area (f).

**Table 1:** Liver function of the mice at each time point after intravenous injection of ConA.

Modeling time (H)	*N*	ALT (U/L)	AST (U/L)	Albumin (*A*) (g/L)	Globulin (*G*) (g/L)	*A*/*G* Ratio (*A*/*G*)
Control	8	38.8 ± 8.9	136 ± 24	26.3 ± 1.4	25.5 ± 1.2	1.02 ± 0.1
2	7	40.7 ± 6.5	170.6 ± 11.7	25.3 ± 1.6	24.6 ± 1.8	1.05 ± 0.1
8	5	208.8 ± 107.5^*∗*^	558.5 ± 180.1^*∗*^	26.5 ± 2.2	28.4 ± 0.7	0.9 ± 0.1
24	7	107.8 ± 41.4^#^	259.7 ± 78.6^#^	21.5 ± 8.3	30.2 ± 10.1	0.74 ± 0.2
48	8	68.1 ± 39.2	188 ± 59.4	21.9 ± 6.7	30.9 ± 9.7	0.68 ± 0.2

^*∗*^ALT and AST levels were significantly increased on 8 h after ConA injection, compared with the control group (*P* all < 0.01); after that time, a decreasing trend was observed,

^#^24 h after ConA injection, ALT and AST levels were still higher than the level of control group (*P* all < 0.05).

**Table 2:** Levels of serum TGF-*β*1 and TGF-*β*3 in mice after ConA injection.

	(*n*)	TGF-*β*1 (pg/mL)	TGF-*β*3 (pg/mL)	TGF-*β*3/*β*1 ratio
Control	8	21.12 ± 4.01	10.56 ± 6.79	0.45 ± 0.12
2 h	7	25.34 ± 6.32	14.32 ± 8.92	0.53 ± 0.23
8 h	5	36.43 ± 9.67^*∗*^	25.34 ± 6.45^#^	0.69 ± 0.09
24 h	7	28.77 ± 8.44^*∗*^	18.37 ± 11.25^#^	0.62 ± 0.18
48 h	8	25.79 ± 9.24	9.26 ± 3.14	0.35 ± 0.06^*∗*#^

^*∗*^8 h and 24 h after ConA injection, serum concentrations of TGF-*β*1 were significantly higher than the control group (*P* = 0.031 and 0.046, resp.); peak time was 8 h.

^#^After ConA injection, on 8 h and 24 h, concentration of TGF-*β*3 was also significantly higher than the control group (*P* = 0.025 and 0.043, resp.); peak time was 8 h.

^*∗*#^After ConA injection, the ratio of TGF-*β*3/TGF-*β*1 decreased significantly at 48 h compared with control group (*P* = 0.046).

**Table 3:** Pearson's correlation between TGF-*β*1, TGF-*β*3, ratio of TGF-*β*3/TGF-*β*1, liver function, and *α*-SMA in liver of mice in each group.

Correlations	TGF-*β*1	TGF-*β*3	Ratio	ALT	AST	Globulin	SMA
TGF-*β*1							
Pearson's correlation	1	.918^*∗*^	.770	.967^*∗∗*^	.966^*∗∗*^	.388	.918^*∗*^
Sig. (2-tailed)		.028	.128	.007	.007	.518	.028
*N*	5	5	5	5	5	5	5
TGF-*β*3							
Pearson's correlation	.918^*∗*^	1	.956^*∗*^	.907^*∗*^	.918^*∗*^	.117	.731
Sig. (2-tailed)	.028		.011	.033	.028	.851	.160
*N*	5	5	5	5	5	5	5
Ratio							
Pearson's correlation	.770	.956^*∗*^	1	.756	.764	−.040	.556
Sig. (2-tailed)	.128	.011		.139	.133	.949	.330
*N*	5	5	5	5	5	5	5
ALT							
Pearson's correlation	.967^*∗∗*^	.907^*∗*^	.756	1	.987^*∗∗*^	.405	.869
Sig. (2-tailed)	.007	.033	.139		.002	.499	.056
*N*	5	5	5	5	5	5	5
AST							
Pearson's correlation	.966^*∗∗*^	.918^*∗*^	.764	.987^*∗∗*^	1	.275	.819
Sig. (2-tailed)	.007	.028	.133	.002		.654	.090
*N*	5	5	5	5	5	5	5
Globulin							
Pearson's correlation	.388	.117	−.040	.405	.275	1	.705
Sig. (2-tailed)	.518	.851	.949	.499	.654		.183
*N*	5	5	5	5	5	5	5
SMA							
Pearson's correlation	.918^*∗*^	.731	.556	.869	.819	.705	1
Sig. (2-tailed)	.028	.160	.330	.056	.090	.183	
*N*	5	5	5	5	5	5	5

^*∗*^Correlation is significant at the 0.05 level (2-tailed).

^*∗∗*^Correlation is significant at the 0.01 level (2-tailed).

Ratio: TGF-*β*3/TGF-*β*1.
